# Is Early Tobacco–Rice Rotation Associated with Soil Nutrient Legacy and Shifts in Bacterial Ecological Potential in a Subtropical Paddy System?

**DOI:** 10.3390/plants15142230

**Published:** 2026-07-21

**Authors:** Xiang-Bin Yao, Yong-Yu Liang, Ya-Jun Cheng, Chun-Yan Li, Jie-Yu-Xin Kang, Jian-Ying Qi

**Affiliations:** College of Agriculture, South China Agricultural University, 483 Wushan Road, Guangzhou 510642, China; yaoxbgd23@stu.scau.edu.cn (X.-B.Y.);

**Keywords:** tobacco–rice rotation, soil nutrient legacy, bacterial community, predicted functional potential, paddy soil, late-season rice

## Abstract

In subtropical double-rice regions, replacing early-season rice with tobacco may alter the soil environment inherited by subsequent paddy cultivation. We conducted a one-year field experiment in 2023 to compare rice–rice (RR) and tobacco–rice (TR) pathways. Soils from the 0–10 and 10–20 cm layers were sampled during the early-season pathway-differentiation period and the late-season rice-growing period, and soil properties, bacterial 16S rRNA communities, predicted functional profiles, and rice agronomic traits were analyzed. Relative to RR, TR was associated with 4.12% lower grain yield but 5.10% higher 1000-grain weight and 23.74% higher aboveground dry matter, indicating altered yield formation rather than uniform growth suppression. TR was also associated with lower soil organic carbon (SOC), total nitrogen (TN), alkali-hydrolyzable nitrogen (AN), and available phosphorus (AP). Bacterial communities differed between pathways, with stronger *Chloroflexi*/*Actinobacteriota* and 16S-predicted aerobic, stress-tolerant, and chemoheterotrophic signals under TR. Together, these findings indicate that early TR was associated with lower measured C-, N-, and P-related soil pools, bacterial community reorganization, and altered late-season rice yield formation; therefore, nutrient and residue management should be planned across the tobacco–rice year rather than only during the rice season.

## 1. Introduction

Subtropical rice-based farming systems are being adjusted rapidly as farmers seek higher land-use efficiency, more diverse income sources, and more flexible seasonal production [1]. In double-rice regions of southern China, one important change is the partial replacement of the traditional rice–rice (RR) pathway by rotations in which an upland crop occupies the early season and rice is cultivated later [2]. This pathway change is agronomically attractive because it can diversify production and improve seasonal land use, but it may also modify the soil environment in which late-season rice begins growth. The early-season crop contributes to residue input, fertilizer demand, nutrient removal, aeration status, water regime, and disturbance timing before rice transplanting [3]. For soil science and plant nutrition, the key issue is therefore not only whether crop diversification is associated with yield, but whether the preceding crop leaves a soil nutrient legacy signal relevant to the following paddy phase.

The relationship between a preceding upland crop and the following rice crop is also a question of temporal nutrient coupling. Fertilizer applied to the upland crop, nutrients removed in harvested biomass, and residues returned or removed before flooding together contribute to the resource state inherited by late-season rice [3]. In practice, farmers often manage the two crop phases as separate production units, but soil processes integrate them across the whole rotation year. A reduction in SOC, TN, AN, or AP after the upland phase may influence rice even when the rice-season fertilization rate appears adequate [3]. Conversely, if the upland phase improves substrate diversity or stimulates beneficial microbial turnover, it may generate a favorable legacy signal [4]. These contrasting possibilities make early-stage field evidence necessary.

A further reason to examine TR early is that management decisions are made before long-term soil changes become visible. If SOC or nutrient availability declines over multiple years, recovery may require sustained amendment inputs [5]. Early diagnosis can therefore indicate whether residue return, organic inputs, balanced fertilization, or subsurface monitoring should be introduced during pathway establishment. Accordingly, this study evaluates whether soil nutrient pools, bacterial community structure, 16S-predicted functional potential, and rice yield components show the same RR–TR contrast during early TR establishment.

The focus on both sampling period and soil depth is also essential [6]. Early-season samples capture the direct divergence between the upland and paddy pathways, whereas late-season samples indicate whether those differences remain after rice cultivation resumes. The 0–10 cm layer is most exposed to residue input and disturbance, while the 10–20 cm layer may reveal a more persistent soil legacy [6]. Considering both dimensions reduces the risk of interpreting a temporary surface signal as a whole-profile response.

Tobacco–rice (TR) rotation provides a useful system for examining this question because tobacco and rice differ strongly in cultivation environment and nutrient-use pattern [2]. Tobacco is generally grown under upland conditions, whereas rice is managed under flooded or periodically flooded conditions [7]. The transition from upland tobacco to flooded rice therefore represents a change from a more oxygenated soil environment to a more reducing paddy environment. Such a transition can influence organic matter turnover, nitrogen mineralization and immobilization, phosphorus availability, potassium cycling, and the balance between nutrient input and crop nutrient export [8]. Even if late-season rice receives the same water, fertilizer, and field management after transplanting, the soil inherited from the early-season crop may differ in carbon and nutrient pools. This inherited soil condition can constrain or support rice growth during tillering, panicle initiation, grain filling, and dry matter accumulation [9].

A soil nutrient legacy is especially relevant during the early establishment of a new rotation pathway. Long-term rotations may eventually develop relatively stable soil biological and physicochemical states, but the first years after pathway divergence can reveal the direction of change before a new equilibrium is reached. If TR is associated with lower soil organic carbon (SOC), total nitrogen (TN), alkali-hydrolyzable nitrogen (AN), or available phosphorus (AP), late-season rice may enter its reproductive period under a lower plant-nutrition background than rice grown after early-season rice [3]. Conversely, if crop diversification is associated with greater residue diversity or improved microbial activity, the rotation may enhance nutrient cycling [4]. Distinguishing between these possibilities is important because rice yield is determined not only by current-season fertilizer input but also by the capacity of soil to buffer nutrient demand during tillering, panicle formation, grain filling, and dry matter accumulation [9].

Soil bacteria provide a sensitive biological layer for detecting these early pathway-associated signals [10]. Bacterial communities respond to soil pH, carbon availability, nutrient status, redox condition, root input, and water regime, and they contribute to organic matter decomposition, nitrogen transformation, phosphorus mobilization, methane-related processes, and other paddy-field functions [11,12]. In flooded rice soils, anaerobic and facultatively anaerobic groups are often favored by reducing conditions, whereas upland phases can select for taxa associated with aerobic metabolism, stress tolerance, and decomposition of more oxidized organic substrates [13]. Thus, bacterial community composition may carry information about soil conditions associated with the preceding crop and rice-root context [14]. When soil nutrient pools shift together with bacterial community structure, the combined evidence is stronger than either soil chemistry or community composition alone.

At the same time, functional interpretation of bacterial community data requires caution. Amplicon sequencing of the 16S rRNA gene can describe taxonomic patterns and diversity, but it does not directly measure gene abundance, transcript activity, enzyme rates, nutrient transformation, or greenhouse gas fluxes [15]. Tools such as BugBase, FAPROTAX, and Tax4Fun infer bacterial phenotypes or functional potential from taxonomic information and reference databases [16,17,18]. These predictions are useful for ecological screening because they can indicate whether community shifts are consistent with aerobic, anaerobic, chemoheterotrophic, phototrophic, or stress-related signals. However, they should be interpreted as predicted functional potential rather than measured function [15]. A defensible manuscript should therefore connect predicted functional profiles to measured soil properties and crop traits, while avoiding claims that the rotation directly changed process rates unless those rates were measured.

Previous studies have shown that crop rotation can increase microbial diversity, improve belowground community functions, and change soil organic matter dynamics in agroecosystems [4]. Work on paddy–upland systems has also demonstrated that conversion between flooded rice and upland crops can alter soil and root-associated microbial communities [2]. However, several gaps remain for TR systems. First, most studies emphasize established rotations or broad paddy–upland comparisons, whereas early-stage divergence from a shared cropping history is less frequently examined. Second, soil nutrient pools, bacterial composition, and predicted functional potential are often studied separately, making it difficult to identify whether microbial shifts correspond to resource differences relevant to plant nutrition. Third, soil layers are not always considered, even though surface soil may respond more quickly to residue input and disturbance while deeper soil may retain a more persistent legacy. Fourth, the link between soil-microbial reorganization and late-season rice yield components remains insufficiently resolved.

Addressing these gaps matters for both scientific interpretation and field management. If early TR establishment is associated mainly with changes in rice yield components but not with altered soil nutrient pools, management could focus on cultivar selection and seasonal agronomy. If, however, TR is associated with a persistent decline in SOC, TN, AN, and AP, then the rotation should be managed as a soil fertility issue as well as a crop-diversification strategy. In that case, residue return, fertilizer adjustment, and soil fertility monitoring would become central to maintaining late-season rice productivity [3]. Similarly, if bacterial community shifts are associated with lower nutrient availability and 16S-predicted aerobic or stress-tolerant signals, the microbial response may help identify the ecological direction of pathway change [15]. The integrated analysis therefore tests whether lower nutrient pools, altered bacterial composition, predicted functional signals, and yield-component changes occur in the same pathway direction, which is more informative for TR management than examining yield or microbial composition alone.

Accordingly, this study aimed to determine whether early establishment of the TR pathway, relative to RR, was associated with (1) altered late-season rice yield formation, (2) a detectable soil nutrient legacy signal, particularly in SOC, TN, AN, and AP, and (3) directional shifts in bacterial community composition and 16S-predicted functional potential. We further evaluated whether lower C-, N-, and P-related soil pools, bacterial reorganization, and yield-component changes occurred concurrently during the tobacco–rice year.

## 2. Materials and Methods

### 2.1. Study Site, Cropping History, and Experimental Design

The one-year field experiment was conducted in 2023 in Nanxiong City, Guangdong Province, China (25.15311730° N, 114.37274087° E; 135 m a.s.l.), a subtropical monsoon region with clay-dominated topsoil. The field plots had a shared cropping history before pathway divergence in 2023. Two rotation pathways were compared: RR (rice–rice) and TR (tobacco–rice). Each pathway contained three field replicates arranged in a randomized block design, and each field plot was 200 m^2^. The field replicate was treated as the experimental unit for pathway-level interpretation. Quadrats, composite soil samples, and sequencing samples were considered measurement or profiling units within this pathway comparison and were not used to inflate the number of independent field replicates. The basic physicochemical properties of the experimental soil were as follows: pH 5.85, 0.83 g kg^−1^ total nitrogen (TN), 0.55 g kg^−1^ total phosphorus (TP), 10.90 g kg^−1^ total potassium (TK), and 20.54 g kg^−1^ soil organic carbon (SOC). Because formal pre-differentiation baseline soil measurements were not available, pathway differences are interpreted as pathway-associated responses during early establishment rather than as definitive long-term causal effects.

The late-season rice variety used for agronomic evaluation was the fragrant rice cultivar 19xiang. Under the RR pathway, early-season rice was sown in March and harvested in July, and late-season rice was sown in July and harvested in November. Under the TR pathway, the upland preceding crop (tobacco) occupied the early-season period from February to July, followed by late-season rice from July to November.

### 2.2. Rice Cultivation and Field Management

Except for the early-season pathway, RR and TR received the same late-season rice management. Seeds were soaked for 24 h and germinated at 34 °C for 12 h before tray sowing, following common rice seedling management principles [9]. Seedlings were mechanically transplanted at approximately 15 d after sowing. Late-season rice was sown, transplanted, and harvested on 15 July, 30 July, and 3 November respectively, at 14 cm × 30 cm spacing.

A fragrant-rice compound fertilizer (Dongguan Fute Biotechnology Co., Ltd., Dongguan, China) was applied as basal and tillering fertilizer at 900 kg ha^−1^, supplying 135 kg ha^−1^ N, 36 kg ha^−1^ P_2_O_5_, and 54 kg ha^−1^ K_2_O. The basal and tillering fertilizer portions were applied at a ratio of 5:2. Basal fertilizer was mechanically side-deep applied at transplanting, whereas tillering fertilizer was surface-applied during tillering, consistent with the need to match rice nutrient supply to growth-stage demand [3]. Water, weed, pest, and disease management followed local high-yield practices and were consistent between pathways.

Tobacco seedlings were raised in a nursery substrate consisting of 60% field soil and 40% fully composted pig manure and were transplanted under plastic mulch at 18,000–19,500 plants ha^−1^. Fertilization was basal-dominant, with 45–90 kg N ha^−1^ applied according to soil fertility and an N:P:K ratio of 1:2:3. Irrigation was supplied during critical water-demand periods, while drainage ditches were maintained to prevent waterlogging. Intertillage and earthing-up were conducted as required, and topping and sucker removal were performed during plant development. Pest and disease management combined preventive measures, chemical control, and manual removal.

### 2.3. Measurement of Agronomic Traits of Late-Season Rice

At maturity of late-season rice, grain yield and yield components were measured using quadrat harvesting, a common approach for field-based rice yield assessment [19]. Four 1.0 m^2^ yield-measurement quadrats were established within each rotation pathway to estimate pathway-level yield and yield components, and grain yield was adjusted to a moisture content of 14%. Because these quadrats were measurement subsamples rather than independent field blocks, agronomic comparisons are interpreted as processed quadrat-level estimates supporting pathway-level interpretation.

Effective panicles were recorded before harvest and converted to panicle number per unit area. Panicle number was recorded from 15 observations per pathway. Four representative plants were randomly selected to determine spikelets per panicle, seed-setting rate, and 1000-grain weight. Aboveground dry matter was determined by whole-plant sampling after heating at 105 °C for 30 min and oven-drying at 80 °C to constant weight [9]. Outlier handling was applied only to the agronomic table. Values identified during data checking as recording or measurement outliers were excluded before summary statistics. When a single value was removed from a pathway–trait combination, the missing value was imputed using the mean of the remaining values in the same pathway–trait combination. The excluded values and imputation status are documented in the agronomic processing note in the data archive.

### 2.4. Soil Sampling and Physicochemical Measurements

#### 2.4.1. Soil Sampling

Soil samples were collected at two key periods: the early-season pathway-differentiation period on 10 June 2023 and the late-season rice-growing period on 29 October 2023. At each period, samples were collected from the 0–10 and 10–20 cm layers to capture surface and subsurface responses to rotation management [6]. For soil physicochemical measurements, three field-replicate composite samples were analyzed for each pathway–period–layer combination (2 pathways × 2 periods × 2 depths × 3 field replicates = 24 composite samples). For 16S rRNA sequencing, six sequencing samples were available for each pathway–period–layer combination (2 pathways × 2 periods × 2 depths × 6 samples = 48 sequencing samples); these sequencing samples were used for community profiling and were not interpreted as additional independent field plots. After the documented multimetric filtering step, five sequencing profiles per pathway–period–layer combination were retained for the alpha-diversity analyses summarized in Supplementary Table S4. Accordingly, community-level analyses were interpreted as sample-level community patterns nested within the pathway comparison rather than as additional field-block replication.

Sampling was performed using a soil auger, following standard soil sampling and handling principles [20]. After collection, plant residues and gravel were removed, and each sample was thoroughly mixed and divided into two portions. One portion of fresh soil was sieved and stored at −80 °C for DNA extraction and microbial analysis to preserve microbial DNA integrity [21]. The other portion was air-dried, ground, and sieved for the determination of soil physicochemical properties [22].

#### 2.4.2. Determination of Soil Physicochemical Properties

Air-dried soil samples were ground and passed through a 0.25 mm sieve for the determination of alkali-hydrolyzable nitrogen (AN), available phosphorus (AP), available potassium (AK), soil organic carbon (SOC), and total nitrogen (TN). All reagents used for soil physicochemical analyses were of analytical grade and were purchased from Shanghai Aladdin Biochemical Technology Co., Ltd. (Shanghai, China). AN was determined by alkaline hydrolysis diffusion [22]. AP was measured by sodium bicarbonate extraction followed by molybdenum–antimony colorimetry using an Epoch microplate spectrophotometer (BioTek Instruments, Inc., Winooski, VT, USA) [23,24]. AK was determined by extraction with 1 mol L^−1^ ammonium acetate (NH_4_OAc), followed by flame atomic absorption spectrometry using an AA-6300C atomic absorption spectrophotometer (Shimadzu Corporation, Kyoto, Japan) [25]. Before SOC measurement, inorganic carbon was removed using 1 mol L^−1^ hydrochloric acid (HCl). The samples were rinsed with deionized water, dried, and SOC and TN were determined simultaneously by high-temperature combustion with thermal conductivity detection using a vario MACRO cube elemental analyzer (Elementar Analysensysteme GmbH, Langenselbold, Germany) [26,27].

### 2.5. DNA Extraction, 16S rRNA Sequencing, and Bioinformatic Analysis

Soil bacterial community diversity was characterized using 16S rRNA gene amplicon sequencing [28]. After extraction of soil genomic DNA, DNA quality was checked by 1% agarose gel electrophoresis [21]. The V3–V4 region of the bacterial 16S rRNA gene was amplified using barcode-tagged primers 338F (5′-ACTCCTACGGGAGGCAGCAG-3′) and 806R (5′-GGACTACHVGGGTWTCTAAT-3′) [29]. PCR products were purified, quantified, pooled in equimolar amounts, and used for Illumina library construction. Paired-end sequencing was performed on an Illumina NovaSeq 6000 platform (Illumina, Inc., San Diego, CA, USA) by Majorbio Bio-Pharm Technology Co., Ltd. (Shanghai, China).

After sample demultiplexing, PE reads obtained from Illumina sequencing were assigned to samples based on barcode and primer sequences. Raw sequences were quality-filtered using Trimmomatic (v0.33) and merged using FLASH (v1.2.11) [30,31]. After quality control and merging, a total of 3,285,391 optimized sequences were obtained from 48 soil samples, with 1,365,931,593 total bases and an average sequence length of 415 bp.

Optimized sequences were clustered into operational taxonomic units (OTUs) at 97% similarity using USEARCH v11.0, with chimera removal during clustering [32,33]. Good’s coverage ranged from 0.945 to 0.978 across the 48 samples, indicating adequate sequencing coverage for the processed bacterial community profiles. OTU representative sequences were taxonomically annotated using QIIME (v1.9.1) with the RDP Classifier or NCBI BLAST (https://blast.ncbi.nlm.nih.gov/Blast.cgi, accessed on 19 July 2026) against the SILVA database (Release 138) [34,35,36]. The available sequencing summary did not provide a separate count for chloroplast or mitochondrial read removal, so no unsupported removal percentage is reported here. Alpha diversity was calculated using mothur v1.30.1 [37,38], and beta diversity was evaluated using Bray–Curtis distances, non-metric multidimensional scaling (NMDS) ordination, and the statistical procedures described in Section 2.6 [39,40]. BugBase (https://bugbase.cs.umn.edu/), FAPROTAX v1.2.1, and Tax4Fun v0.3.1 were used only to infer bacterial phenotypes and 16S-predicted functional potential [15,16,17,18].

### 2.6. Statistical Analysis

For agronomic traits, *p* values were calculated from the processed quadrat-level table and interpreted with the sampling hierarchy described in Section 2.3; they should therefore be treated as processed quadrat-level comparisons supporting pathway-level interpretation rather than as independent field-block tests. Continuous variables were examined for distributional assumptions and homogeneity of variance before significance testing [41,42]. When assumptions were met, pathway differences were evaluated by analysis of variance followed by the least significant difference test; otherwise, Mann–Whitney U or Kruskal–Wallis tests were used [19,43,44]. For bacterial beta diversity, Bray–Curtis distance matrices were used for non-metric multidimensional scaling (NMDS) ordination, analysis of similarities (ANOSIM), permutational multivariate analysis of variance (PERMANOVA), and the permutational test for homogeneity of multivariate dispersions (PERMDISP; implemented using betadisper) [39,45]. PERMANOVA was used to test differences in bacterial community composition among pathway–period groups, whereas PERMDISP was used to examine the homogeneity of multivariate dispersion among groups. These analyses were conducted separately for the 0–10 cm and 10–20 cm soil layers, consistent with the NMDS panels. Multiple-feature comparisons were corrected by the Benjamini–Hochberg method [46]. Soil–community–function associations were assessed using redundancy analysis (RDA), Spearman correlation, Mantel tests, and variance partitioning analysis (VPA) [40,47,48]. Significance was set at *p* < 0.05.

## 3. Results

### 3.1. Agronomic Traits of Late-Season Rice

Compared with RR, TR had 4.12% lower late-season grain yield, and this difference was significant in the processed quadrat-level comparison (Table 1). Seed-setting rate did not differ significantly between the two pathways.

TR had 8.74% higher effective panicle number, although this difference was not significant. In contrast, 1000-grain weight and aboveground dry matter were significantly higher under TR by 5.10% and 23.74%, respectively, in the processed quadrat-level comparison. Spikelets per panicle and seed-setting rate decreased by 5.15% and 1.33%, respectively, but neither difference was significant. The grain-yield-to-dry-matter ratio was lower under TR (0.366) than under RR (0.472), corresponding to an approximately 22.4% reduction. Thus, higher biomass and grain weight under TR did not translate into higher grain yield, and the lower grain-yield-to-dry-matter ratio indicates altered biomass partitioning toward non-grain dry matter.

### 3.2. Soil Physicochemical Properties

**Figure 1:**
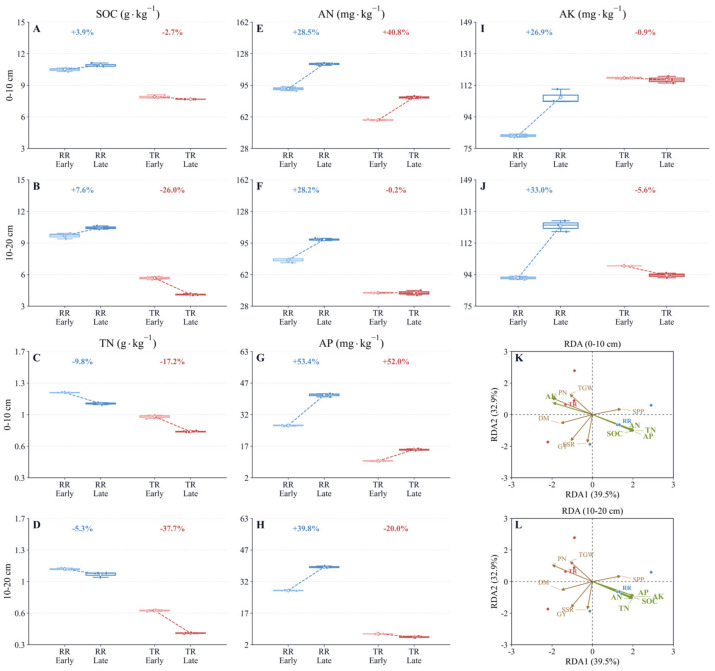
Effects of rotation pathway on soil properties and their associations with late-season rice agronomic traits. (**A**,**B**) SOC, (**C**,**D**) TN, (**E**,**F**) AN, (**G**,**H**) AP, and (**I**,**J**) AK in the 0–10 and 10–20 cm soil layers, respectively, during the Early and Late periods. Points represent replicates; boxes show the interquartile range and median; whiskers extend to 1.5 × the interquartile range; diamonds indicate means; and dashed lines connect Early and Late means. Percentages indicate Early-to-Late changes in the mean. (**K**,**L**) RDA biplots for the two soil layers. Green and brown arrows represent soil properties and agronomic traits, respectively; arrow direction and length indicate the direction and strength of association. RR, rice–rice; TR, tobacco–rice; SOC, soil organic carbon; TN, total nitrogen; AN, alkali-hydrolyzable nitrogen; AP, available phosphorus; AK, available potassium; RDA, redundancy analysis; GY, grain yield; DM, aboveground dry matter; PN, panicle number; SPP, spikelets per panicle; TGW, 1000-grain weight; SSR, seed-setting rate.

SOC, TN, AN, and AP were generally higher under RR than under TR across both sampling periods and soil layers (Figure 1A–J). Thus, the TR pathway had lower measured organic C, total and alkali-hydrolyzable N, and available P pools than RR at the sampled stages. In contrast, AK responses were inconsistent and varied with sampling period and soil depth.

Late-season RR and TR samples were separated in ordination space in both soil layers (Figure 1K,L). In the 0–10 cm layer, TN, SOC, AN, and AP were oriented in the same direction as spikelets per panicle but in the opposite direction to effective panicle number, 1000-grain weight, and aboveground dry matter. A broadly similar pattern was observed in the 10–20 cm layer, where AK tended to align more closely with the other soil variables. Grain yield and seed-setting rate showed weaker associations with the measured soil factors.

### 3.3. Bacterial Alpha Diversity

**Figure 2:**
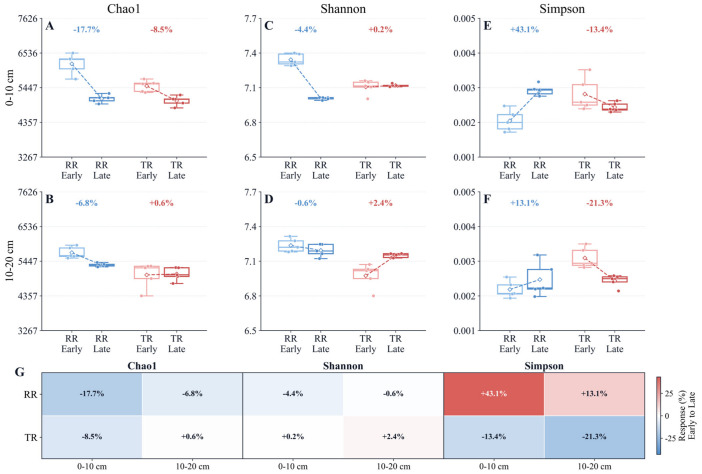
Responses of bacterial alpha diversity to rotation pathway, sampling period, and soil depth. (**A**,**B**) Chao1 richness, (**C**,**D**) Shannon diversity, and (**E**,**F**) Simpson indices in the 0–10 and 10–20 cm soil layers, respectively. Points represent samples; boxes show the interquartile range and median; whiskers extend to 1.5 × the interquartile range; diamonds indicate means; and dashed lines connect Early and Late means. Percentages indicate Early-to-Late changes in the mean. (**G**) Heatmap of the corresponding percentage changes, with red indicating increases and blue indicating decreases. RR, rice–rice; TR, tobacco–rice. Each pathway–period–depth group contains n = 5 profiles after the multimetric filtering step.

Chao1, Shannon, and Simpson indices showed period- and depth-dependent responses to rotation pathway (Figure 2A–F). Chao1 was generally higher under RR than under TR, especially during early-season pathway differentiation. After late-season rice cultivation, this difference narrowed in the 0–10 cm layer but remained more evident at 10–20 cm. Shannon and Simpson showed smaller and directionally variable responses across periods and depths.

The response heatmap showed that from the early-season pathway-differentiation period to the late-season rice-growing period, Chao1 and Shannon generally decreased under RR, whereas Simpson increased. Under TR, Chao1 decreased less strongly, Shannon remained stable or slightly increased, and Simpson generally decreased (Figure 2G). Thus, alpha diversity did not show a single RR–TR direction; its pathway response depended on sampling period, soil depth, and the diversity index considered.

### 3.4. Separation of Bacterial Community Structure

**Figure 3:**
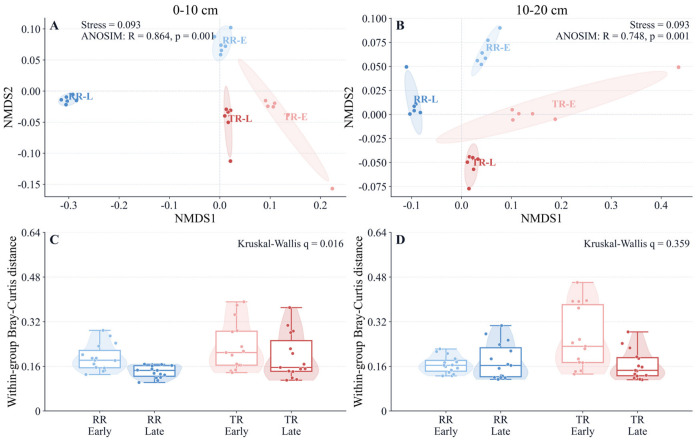
Effects of rotation pathway on bacterial beta diversity and community dispersion. (**A**,**B**) NMDS ordination based on Bray–Curtis dissimilarities in the 0–10 and 10–20 cm soil layers. Points represent samples and shaded ellipses indicate group dispersion. Stress indicates ordination fit, and ANOSIM R indicates the strength of group separation. (**C**,**D**) Within-group Bray–Curtis dissimilarities; violin shapes show distributions, boxes show the interquartile range and median, and points show individual pairwise distances. q denotes the Benjamini–Hochberg-adjusted Kruskal–Wallis *p* value. RR, rice–rice; TR, tobacco–rice; NMDS, non-metric multidimensional scaling; ANOSIM, analysis of similarities.

NMDS ordination based on Bray–Curtis distances showed separation among pathway–period groups in both soil layers, with stronger separation in the 0–10 cm layer than in the 10–20 cm layer (Figure 3A,B). PERMANOVA confirmed significant differences in bacterial community composition among groups in both soil layers (0–10 cm: F = 11.6305, R^2^ = 0.6356, *p* = 0.001; 10–20 cm: F = 6.6286, R^2^ = 0.4986, *p* = 0.001). ANOSIM produced consistent results (0–10 cm: R = 0.864, *p* = 0.001; 10–20 cm: R = 0.748, *p* = 0.001). In contrast, PERMDISP was not significant in either soil layer (0–10 cm: F = 1.6449, *p* = 0.357; 10–20 cm: F = 2.2896, *p* = 0.226), indicating that the observed community separation was not mainly driven by unequal within-group dispersion.

Within-group Bray–Curtis distances showed visible variation among groups, especially in the 0–10 cm layer, where TR samples tended to be more dispersed than RR samples during the early season and late-season RR showed stronger within-group clustering. However, PERMDISP did not detect significant heterogeneity of multivariate dispersion in either soil layer, so the NMDS separation was interpreted mainly as a difference in community composition rather than as an artifact of unequal dispersion (Figure 3C,D).

### 3.5. Differences in Late-Season Community Composition

**Figure 4:**
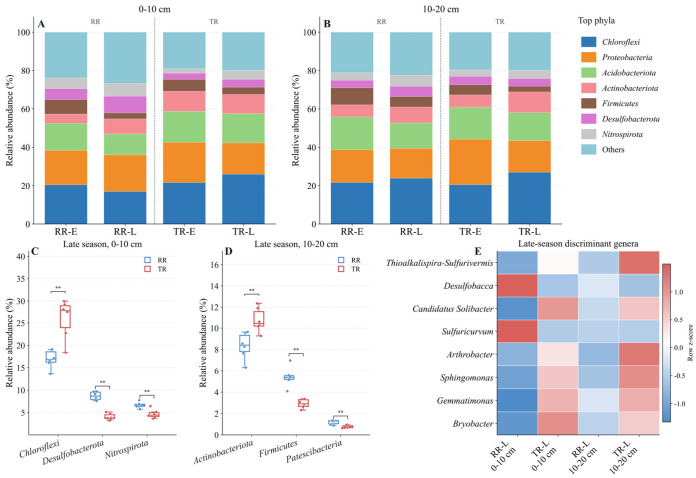
Bacterial community composition and discriminant taxa under different rotation pathways. (**A**,**B**) Relative abundance of dominant bacterial phyla in the 0–10 and 10–20 cm soil layers; Others includes phyla not displayed separately. (**C**,**D**) Late-season abundances of selected phyla. Points represent samples; boxes show the interquartile range and median; whiskers extend to 1.5 × the interquartile range. (**E**) Row-standardized heatmap of the eight interpretable genera with the largest descriptive RR–TR mean-abundance differences. Red and blue indicate values above and below the row mean, respectively. These genera were descriptively ranked rather than selected by a formal differential-abundance test. RR, rice–rice; TR, tobacco–rice; ** *p* < 0.01.

RR and TR shared broadly similar dominant bacterial phyla across periods and soil layers, but their relative abundance distributions differed (Figure 4A,B). Therefore, pathway-associated differences were expressed mainly as shifts in community composition rather than replacement of dominant phyla.

Late-season taxonomic differences varied by soil depth. In the 0–10 cm layer, TR showed a higher relative abundance of *Chloroflexi*, whereas RR showed higher relative abundances of *Desulfobacterota* and *Nitrospirota*. In the 10–20 cm layer, TR showed a higher relative abundance of *Actinobacteriota*, whereas *Firmicutes* and *Patescibacteria* accounted for higher proportions under RR (Figure 4C,D).

The discriminant-genus heatmap further showed that pathway separation was also visible at the genus level. Compared with RR, genera such as *Arthrobacter*, *Sphingomonas*, and *Gemmatimonas* were relatively more abundant under TR, whereas *Desulfobacca* and *Sulfuricurvum* showed higher relative abundances under RR (Figure 4E). Therefore, the pathway contrast was not restricted to phylum-level shifts, although these genera should be treated as compositional markers rather than direct functional evidence.

### 3.6. Shifts in Predicted Phenotypes and Functional Potential

**Figure 5:**
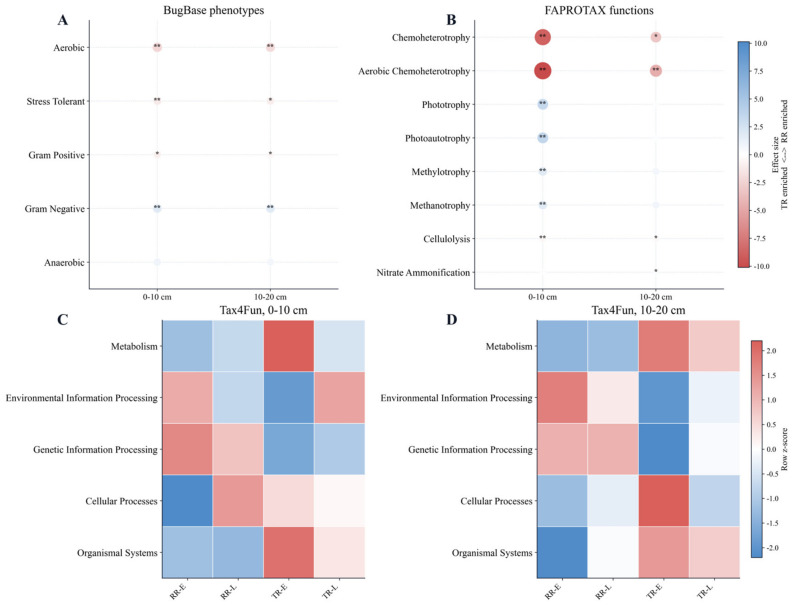
Bacterial phenotypes and functional potential predicted from 16S rRNA gene data. (**A**) BugBase-predicted phenotypes and (**B**) FAPROTAX-predicted functions during the Late period. Bubble size indicates the absolute effect size; red indicates enrichment under TR and blue indicates enrichment under RR. (**C**,**D**) Row-standardized Tax4Fun level-1 pathway profiles in the 0–10 and 10–20 cm soil layers. Red and blue indicate values above and below the row mean, respectively. These tools predict functional potential rather than directly measured microbial activity. RR, rice–rice; TR, tobacco–rice; FAPROTAX, Functional Annotation of Prokaryotic Taxa; * *p* < 0.05, ** *p* < 0.01.

BugBase, FAPROTAX, and Tax4Fun analyses suggested that RR and TR differed in 16S-predicted bacterial phenotypes and functional profiles (Figure 5). Because these tools infer function from taxonomic profiles, Figure 5 is used here to compare predicted ecological tendencies between pathways, not measured gene abundance, enzyme activity, or process rates.

BugBase predictions showed higher aerobic, stress-tolerant, and Gram-positive signals under TR in both soil layers, whereas RR had higher anaerobic and Gram-negative signals (Figure 5A). FAPROTAX predictions suggested that TR was more associated with chemoheterotrophy and aerobic chemoheterotrophy, while RR retained stronger signals related to phototrophy, photoautotrophy, and selected methylotrophy or methanotrophy categories. These contrasts were more evident in the 0–10 cm layer (Figure 5B).

Tax4Fun level-1 profiles also differed between pathways. TR showed stronger predicted metabolism-related signals, whereas RR showed relatively stronger predicted environmental information processing and genetic information processing signals, particularly in the 10–20 cm layer (Figure 5C,D).

### 3.7. Soil–Community–Function Associations

**Figure 6:**
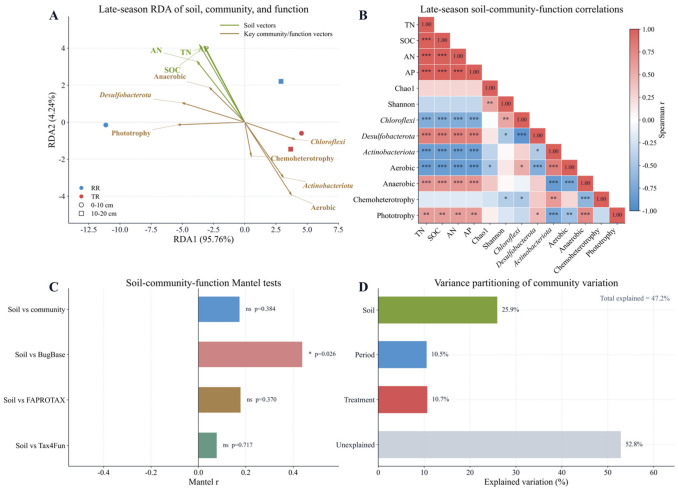
Associations among soil properties, bacterial communities, and predicted functional potential. (**A**) Late-season RDA. Blue and red represent RR and TR; circles and squares represent the 0–10 and 10–20 cm layers; larger symbols indicate group centroids. Green arrows represent soil properties and brown arrows represent bacterial or predicted functional variables; arrow direction and length indicate the direction and strength of association. (**B**) Spearman correlation matrix, with red and blue indicating positive and negative correlations, respectively. (**C**) Mantel correlations between soil properties and community or predicted functional profiles; bar length indicates Mantel r. (**D**) VPA of community variation attributed to soil, sampling period, and rotation pathway; Unexplained indicates residual variation. RR, rice–rice; TR, tobacco–rice; RDA, redundancy analysis; VPA, variance partitioning analysis; TN, total nitrogen; SOC, soil organic carbon; AN, alkali-hydrolyzable nitrogen; AP, available phosphorus; AK, available potassium; * *p* < 0.05, ** *p* < 0.01, *** *p* < 0.001; ns, not significant.

RDA showed that late-season samples were separated along gradients of soil nutrient variables, dominant community features, and predicted functional profiles (Figure 6A). TN, SOC, AN, and AP were closer to RR samples and clustered with *Desulfobacterota*, anaerobic, and phototrophy signals. TR samples were closer to *Chloroflexi*, *Actinobacteriota*, aerobic, and chemoheterotrophy signals. Thus, the ordination placed the lower-nutrient side of the gradient together with TR-enriched taxa and 16S-predicted aerobic or heterotrophic potential.

Spearman correlations further supported this association pattern (Figure 6B). Major soil nutrient factors were positively correlated with *Desulfobacterota*, anaerobic, and phototrophy signals, but negatively correlated with *Chloroflexi*, *Actinobacteriota*, and aerobic signals. Chao1 and Shannon showed weaker correlations with soil factors, indicating that taxonomic composition and predicted functional profiles were more closely aligned with soil nutrient variation than alpha-diversity indices in this dataset.

Mantel tests showed that soil differences had the strongest and statistically significant matrix-level correlation with BugBase-predicted phenotypes, whereas correlations with community composition, FAPROTAX-predicted functions, and Tax4Fun-predicted pathways were not significant (Figure 6C). VPA further indicated that soil variables explained more community variation independently than sampling period or pathway identity (Figure 6D). These results identify measured soil variables, especially C-, N-, and P-related pools, as the covariate set most consistently aligned with bacterial differences in this dataset, while also showing that not all predicted functional datasets were significantly coupled with soil variation at the matrix level.

## 4. Discussion

This study evaluated the early divergence between RR and TR pathways from the perspective of soil nutrient legacy, bacterial ecological response, and late-season rice performance. Instead, TR was associated with a mixed agronomic response: grain yield declined modestly, while 1000-grain weight and aboveground dry matter increased. At the soil level, the most consistent signal was lower SOC, TN, AN, and AP under TR across sampling periods and soil layers. At the microbial level, bacterial communities separated between pathways, and TR was characterized by relative enrichment of *Chloroflexi* and *Actinobacteriota* together with aerobic, stress-tolerant, and chemoheterotrophic 16S-predicted signals. Taken together, the data link early TR with three measured or inferred outcomes: lower C-, N-, and P-related soil pools, altered bacterial composition, and a changed pattern of late-season rice yield formation. Because the study was field-based and focused on early establishment, these outcomes are interpreted as pathway-associated evidence rather than as proof of a complete causal mechanism [15].

### 4.1. Rotation Pathway and Yield Formation

The lower late-season rice grain yield under TR indicates that replacing early-season rice with tobacco was not associated with an immediate yield benefit under the management conditions tested. The yield decline was relatively small and was significant in the processed quadrat-level comparison, indicating a potentially agronomically relevant shift that should be interpreted with the sampling hierarchy described in Section 2.3. At the same time, TR showed higher 1000-grain weight and aboveground dry matter, indicating that yield formation did not respond uniformly across components. This pattern is consistent with a shift in the balance among panicle number, grain setting, grain filling, and biomass allocation rather than simple whole-plant suppression. A heavier grain weight can occur when assimilate supply per grain is increased or when competition among grains is reduced, whereas grain yield also depends on the number of productive panicles and filled grains [9]. Thus, the TR pathway was associated with altered yield structure even as total yield declined.

From a plant-nutrition perspective, this response is consistent with a crop entering the late season under a different soil nutrient background. Rice yield formation depends on nutrient availability during vegetative growth and reproductive development. If soil N and P availability are lower under TR, early tillering, panicle formation, or spikelet differentiation may be constrained even when later grain filling is partly compensated by biomass accumulation [3]. The increase in aboveground dry matter under TR shows that plant growth was not uniformly limited, but grain yield depends on how biomass is partitioned into harvestable yield [9]. This helps explain why yield, 1000-grain weight, and dry matter did not move in the same direction. For this dataset, TR assessment should therefore include the grain-yield-to-dry-matter ratio, yield components, soil nutrient pools, and the timing of nutrient supply, rather than final grain yield or biomass alone.

### 4.2. Soil Nutrient Legacy Signals

The clearest soil pattern in this study is the lower SOC, TN, AN, and AP under TR. These indicators represent C-, N-, and P-related resource pools central to paddy fertility. SOC supports soil structure, nutrient retention, microbial substrates, and long-term fertility [5]. TN and AN reflect the N resource base available for mineralization and plant uptake. AP indicates the P pool most relevant to crop acquisition [3]. Their consistent reduction under TR is consistent with an early-stage soil nutrient legacy signal that remained evident into the late-season rice period. Because late-season rice management was shared between pathways, these differences point to conditions inherited from the preceding crop phase rather than to the late-season rice management regime alone.

Several processes may contribute to this nutrient legacy. Upland tobacco cultivation differs from early-season rice in aeration, root residue quality, fertilization schedule, harvest export, and soil disturbance. More oxygenated upland conditions can stimulate organic matter decomposition relative to flooded conditions, potentially reducing SOC if residue return does not compensate for mineralization [49]. Tobacco also has substantial nutrient demand, and removal of tobacco biomass can export nutrients before late-season rice is planted. In contrast, an early-season rice crop maintains a flooded or periodically flooded paddy environment and may leave different residue and root inputs. These differences can alter the balance between nutrient input, transformation, retention, and export [50].

The reductions in SOC, TN, AN, and AP also have implications for soil fertility management. If TR is associated with lower nutrient pools during the early establishment stage, late-season rice may require adjusted fertilization or residue management to maintain yield [3]. This pattern should not be interpreted as evidence that TR is inherently unsuitable, because rotation performance depends on fertilizer rate, residue return, soil type, irrigation, and duration of establishment [51]. However, it suggests that simply replacing early-season rice with tobacco while keeping late-season rice management unchanged may not be sufficient to maintain the same soil resource background. For farmers and agronomists, the practical message is that TR should be accompanied by monitoring of C, N, and P pools rather than evaluated only by seasonal crop output.

The nutrient legacy signal should also be considered in relation to the seasonal separation of management decisions. In many upland–paddy rotations, fertilizer is planned for each crop separately, whereas soil C and nutrient pools respond to the cumulative balance of input, transformation, and export across seasons [50]. The lower SOC, TN, AN, and AP under TR indicate that the rice crop may have inherited a lower resource background established before transplanting. This may help explain why identical late-season rice management did not eliminate pathway-associated differences. A practical implication is that fertilizer recommendations for TR should not be copied directly from RR systems without accounting for the preceding tobacco phase [3].

### 4.3. Soil-Layer Differentiation and Profile Response

The two sampled soil layers showed whether the RR–TR contrast was confined to surface soil or extended into the upper root zone. Surface soil is usually more responsive to residue input, fertilization, root activity, wetting–drying cycles, and cultivation disturbance, whereas the 10–20 cm layer can retain more persistent signals of management history [6]. In this study, pathway-associated differences were detected in both layers, indicating that TR was linked not only to the immediate surface environment but also to the upper soil profile relevant to rice roots. The detection of differences across layers supports the interpretation of a pathway-associated nutrient legacy signal.

Layer-specific responses are important for paddy systems because rice roots explore both surface and subsurface soil, and nutrient availability at different depths can affect tillering, root development, and grain filling [52]. A nutrient decline restricted only to the surface might be partly buffered by deeper soil reserves, whereas a decline across 0–20 cm suggests a broader change in the soil volume supporting rice growth [6]. The stronger surface microbial separation observed in ordination is also reasonable because surface soil receives more direct effects of crop residue, oxygen exposure, and fertilization [53]. Differences detected at 10–20 cm therefore indicate that the RR–TR contrast was not limited to short-lived surface disturbance. Future sampling over greater depths and additional years would be needed to determine whether this profile pattern strengthens, weakens, or stabilizes as the TR pathway matures.

### 4.4. Community Reorganization and Shifts in Predicted Functional Potential

Bacterial community structure differed between RR and TR, indicating that early pathway divergence was accompanied by biological reorganization. The Bray–Curtis ordination pattern is consistent with pathway-associated differences extending beyond a few individual taxa and involving broader changes in community composition. The relative enrichment of *Chloroflexi* and *Actinobacteriota* under TR is consistent with a community shift toward groups often associated with organic matter decomposition, stress tolerance, and more oxygenated or fluctuating soil conditions [54]. In contrast, RR retained stronger association with taxa and predicted signals more typical of flooded paddy environments [13]. This pattern agrees with the basic environmental contrast between an early-season flooded rice phase and an upland tobacco phase.

The alpha-diversity response was less straightforward than the community-composition response. Chao1, Shannon, and Simpson indices varied by period and soil depth, and they did not provide a single consistent signal of pathway effect. This is not unusual in soil microbial ecology because diversity indices summarize richness and evenness but do not fully describe community membership or functional potential [53]. Diversity indices can remain stable even when community membership changes substantially. In this study, beta-diversity separation and taxonomic shifts appeared more informative than alpha diversity for detecting early TR-associated differences. This suggests that pathway divergence was associated with changes in which bacterial groups were favored rather than simply increasing or decreasing overall diversity.

The predicted phenotype and functional profiles further support the interpretation of a shift in ecological strategy. TR was characterized by aerobic, stress-tolerant, and chemoheterotrophic 16S-predicted signals, while RR showed stronger signals linked with anaerobic or paddy-associated conditions. Such results are consistent with the upland tobacco phase exposing soil to more oxygenated conditions and changed carbon substrate inputs before rice flooding resumed [49]. However, these predictions must be interpreted carefully. BugBase, FAPROTAX, and Tax4Fun infer functional potential from taxonomic data and reference information; they do not measure enzyme activity, functional gene abundance, transcript expression, or process rates [15,16,17,18].

Therefore, the functional result of this study is specific but bounded: TR samples carried stronger 16S-predicted aerobic, stress-tolerant, and chemoheterotrophic signals, whereas RR samples carried stronger anaerobic and paddy-associated signals. Because BugBase, FAPROTAX, and Tax4Fun infer these profiles from taxonomic data, the contrasts identify potential ecological directions rather than measured nutrient-transformation or carbon-cycling rates. Direct metagenomic, enzymatic, and biogeochemical measurements would be required to test whether the predicted shifts correspond to realized soil processes [55].

### 4.5. Soil Resource Differences and Linked Soil–Microbial–Crop Responses

The integrated analyses point to soil resource differences as the measured layer most consistently aligned with the TR microbial pattern. In RDA and correlation analyses, lower SOC, TN, AN, and AP were positioned with TR-enriched *Chloroflexi*/*Actinobacteriota* and predicted aerobic or chemoheterotrophic signals, whereas higher nutrient pools aligned more closely with RR and anaerobic or phototrophy signals. VPA also indicated that soil variables explained more community variation independently than sampling period or pathway identity. These results support soil resource filtering as an interpretation of the measured associations, while water regime, oxygen availability, crop roots, residue quality, fertilizer timing, and management history remain possible co-drivers that were not fully separated in this field comparison [49,56].

The same interpretation also connects the microbial and crop results without imposing an unsupported causal chain. The TR pathway combined lower measured C-, N-, and P-related pools, stronger predicted aerobic or chemoheterotrophic microbial signals, and lower grain-yield-to-dry-matter ratio. These patterns show that late-season rice yield formation changed in the same pathway context in which soil nutrient pools and bacterial composition differed. The evidence therefore supports a linked pathway response, not a demonstrated sequence in which tobacco first changed nutrients, nutrients then changed microbes, and microbes then caused the yield response.

Variance partitioning, Mantel tests, RDA, and correlation analyses support this linked-response interpretation but do not isolate mechanism. These methods identify associations and explain variation, but they cannot fully separate tobacco root effects, residue export, fertilizer timing, water regime, soil disturbance, and microbial feedbacks in a field comparison [40]. The study design captures realistic pathway differences, which is valuable for agronomic relevance, but it also means that the pathway effect includes multiple linked components. For this reason, soil resource filtering is presented here as an interpretation of the observed associations rather than as a fully demonstrated mechanism.

### 4.6. Study Boundaries and Management Implications

This study has several boundaries that should guide interpretation. First, it examined the early stage of pathway establishment, not a mature long-term rotation. Differences in SOC, TN, AN, AP, bacterial composition, and 16S-predicted functional profiles were detectable during the first year, but the data do not indicate whether these differences will intensify, stabilize, or weaken over time. Long-term monitoring is needed to determine whether management adjustment can restore nutrient pools or whether TR is associated with persistent soil fertility decline [5]. Second, the field comparison reflects realistic management but cannot isolate every individual factor, such as tobacco root effects, residue removal, fertilizer timing, water regime, or soil disturbance. Third, functional interpretation was based on 16S-derived prediction tools, so direct process measurements remain necessary [15].

These boundaries lead to specific management priorities. Because TR was associated with lower SOC, TN, AN, and AP and a lower grain-yield-to-dry-matter ratio, nutrient management should be planned across the tobacco–rice year rather than only during the late-season rice season [3]. Maintaining SOC, TN, AN, and AP should be a priority through residue return, organic amendments, optimized N and P fertilization, and monitoring of both surface and subsurface soil. Because yield components responded in different directions, management should also consider when nutrients are supplied relative to tillering, panicle formation, and grain filling, not only total fertilizer input [9]. From a research perspective, future studies should combine multi-year field monitoring with metagenomics, functional genes, enzyme assays, greenhouse gas measurements, and direct nutrient transformation rates [15]. Such work would test whether the 16S-predicted functional signals observed here correspond to realized soil processes.

Overall, early TR establishment was associated with a measurable soil nutrient legacy signal and coincident bacterial community reorganization in a subtropical paddy system. The evidence supports one bounded conclusion: in 2023, the tobacco phase was linked with lower measured C-, N-, and P-related pools before and during late-season rice cultivation, and the same pathway showed bacterial compositional shifts and altered yield formation. Therefore, upland–paddy rotations should be evaluated through soil C, N, and P pools, microbial community indicators, and rice yield components together, rather than through crop yield alone.

Future experiments should separate the main components of the pathway effect. Residue return experiments could test whether SOC and TN reductions are driven mainly by biomass export. Fertilizer gradient trials could determine whether additional N or P compensates for the observed yield decline. Metagenomic or qPCR-based assays could verify whether predicted functional changes correspond to genes involved in nitrification, denitrification, methanogenesis, organic matter decomposition, or phosphorus cycling [55,57]. Such work would move the interpretation from pathway association toward process-level evidence while retaining the field relevance of the TR system.

The present findings also show why soil monitoring in TR systems should include both surface and subsurface layers [6]. If only the surface layer is measured, nutrient changes at 10–20 cm may be overlooked. If only yield is measured, shifts in soil fertility and bacterial community structure may be missed until productivity becomes unstable. Monitoring SOC, TN, AN, AP, bacterial community indicators, and yield components together would show whether nutrient depletion, microbial reorganization, and yield-component changes continue to occur in the same pathway direction. This monitoring should be repeated across years because early responses may not represent the final state of the rotation. Some soil properties may recover after management adjustment, while others may accumulate slowly. Multi-year observation would help distinguish transient establishment effects from persistent rotation legacies and would make the management recommendations more robust [5].

## 5. Conclusions

In this one-year field comparison, early establishment of the tobacco–rice (TR) pathway was associated with 4.12% lower late-season grain yield, 5.10% higher 1000-grain weight, 23.74% higher aboveground dry matter, and a lower grain-yield-to-dry-matter ratio than rice–rice (RR). Thus, the agronomic response under TR was not uniform growth suppression; biomass accumulation and grain weight increased, but a smaller share of dry matter was converted into harvested grain.

TR was also associated with lower SOC, TN, AN, and AP in both sampled soil layers and periods, indicating a lower measured organic-C, N, and P resource background entering the late-season rice phase. The same pathway contrast was accompanied by bacterial community separation, enrichment of *Chloroflexi*/*Actinobacteriota* under TR, and stronger 16S-predicted aerobic, stress-tolerant, and chemoheterotrophic signals. Because these functional profiles were inferred from 16S data, they identify potential ecological directions rather than measured process rates. For early-stage TR systems, the results support residue return or organic amendment, balanced N and P replenishment, monitoring of both 0–10 and 10–20 cm layers, and multi-year tracking of soil nutrients, bacterial indicators, and rice yield components.

## Figures and Tables

**Table 1:** Yield and yield components of late-season rice under RR and TR.

Pathway	Grain Yield (t ha^−1^)	Panicle Number (10^4^ ha^−1^)	Spikelets per Panicle	Seed-Setting Rate (%)	1000-Grain Weight (g)	Dry Matter (t ha^−1^)
RR	6.70 ± 0.08 a	360.54 ± 41.62 a	149.88 ± 23.27 a	60.28 ± 2.42 a	20.80 ± 0.24 b	14.20 ± 0.95 b
TR	6.43 ± 0.07 b	392.06 ± 59.60 a	142.16 ± 5.79 a	59.49 ± 4.18 a	21.86 ± 0.40 a	17.57 ± 1.38 a
TR vs. RR (%)	−4.12	+8.74	−5.15	−1.33	+5.10	+23.74
*p* value	0.002	0.113	0.543	0.752	0.004	0.007

Note: RR, rice–rice; TR, tobacco–rice. Values are means ± SD. Different lowercase letters within a trait indicate significant differences between pathways (*p* < 0.05) in the processed quadrat-level comparison.

## Data Availability

Figure source data, processed tabular datasets, and Python scripts (Python v3.12; Python Software Foundation, Wilmington, DE, USA) supporting the analyses reported in this manuscript are available in Figshare at https://figshare.com/s/ee6a443da6cd4157e42a (accessed on 19 July 2026). The archive includes rice agronomic traits, soil physicochemical data, processed bacterial 16S rRNA summary tables, predicted functional-profile tables, file manifests, variable notes, and figure-generation scripts.

## Supplementary Materials

The following supporting information can be downloaded at https://www.mdpi.com/article/10.3390/plants15142230/s1, Table S1. Sampling hierarchy, statistical unit, and inferential scope. Table S2. Beta-diversity ordination fit and global tests by soil depth. Table S3. Agronomic descriptive summary calculated from all recorded observations. Table S4. Alpha-diversity summaries using the 40 sequencing profiles plotted in Figure 2. Table S5. Contrast-ranked genus selection and late-period group means used in Figure 4E.

## References

[1] Li C., Shi L., Wang K., Liu B., Liao J., An Z., Chang S.X. (2025). Crop rotation differentially increases soil bacterial and fungal diversities in global croplands: A meta-analysis. Nat. Commun..

[2] Hou P.F., Chien C.H., Chiang-Hsieh Y.F., Tseng K.C., Chow C.N., Huang H.J., Chang W.C. (2018). Paddy-upland rotation for sustainable agriculture with regards to diverse soil microbial community. Sci. Rep..

[3] Dobermann A., Fairhurst T.H. (2000). Rice: Nutrient Disorders & Nutrient Management.

[4] Tiemann L.K., Grandy A.S., Atkinson E.E., Marin-Spiotta E., McDaniel M.D. (2015). Crop rotational diversity enhances belowground communities and functions in an agroecosystem. Ecol. Lett..

[5] Bünemann E.K., Bongiorno G., Bai Z., Creamer R.E., De Deyn G., de Goede R., Fleskens L., Geissen V., Kuyper T.W., Mäder P. (2018). Soil quality—A critical review. Soil Biol. Biochem..

[6] Jobbágy E.G., Jackson R.B. (2001). The distribution of soil nutrients with depth: Global patterns and the imprint of plants. Biogeochemistry.

[7] Ponnamperuma F.N. (1972). The chemistry of submerged soils. Adv. Agron..

[8] Breidenbach B., Blaser M.B., Klose M., Conrad R. (2016). Crop rotation of flooded rice with upland maize impacts the resident and active methanogenic microbial community. Environ. Microbiol..

[9] Yoshida S. (1981). Fundamentals of Rice Crop Science.

[10] Jansson J.K., Hofmockel K.S. (2020). Soil microbiomes and climate change. Nat. Rev. Microbiol..

[11] Bardgett R.D., van der Putten W.H. (2014). Belowground biodiversity and ecosystem functioning. Nature.

[12] Fierer N., Jackson R.B. (2006). The diversity and biogeography of soil bacterial communities. Proc. Natl. Acad. Sci. USA.

[13] Liesack W., Schnell S., Revsbech N.P. (2000). Microbiology of flooded rice paddies. FEMS Microbiol. Rev..

[14] Edwards J., Johnson C., Santos-Medellín C., Lurie E., Podishetty N.K., Bhatnagar S., Eisen J.A., Sundaresan V. (2015). Structure, variation, and assembly of the root-associated microbiomes of rice. Proc. Natl. Acad. Sci. USA.

[15] Langille M.G.I., Zaneveld J., Caporaso J.G., McDonald D., Knights D., Reyes J.A., Clemente J.C., Burkepile D.E., Vega Thurber R.L., Knight R. (2013). Predictive functional profiling of microbial communities using 16S rRNA marker gene sequences. Nat. Biotechnol..

[16] Louca S., Parfrey L.W., Doebeli M. (2016). Decoupling function and taxonomy in the global ocean microbiome. Science.

[17] Aßhauer K.P., Wemheuer B., Daniel R., Meinicke P. (2015). Tax4Fun: Predicting functional profiles from metagenomic 16S rRNA data. Bioinformatics.

[18] Ward T., Larson J., Meulemans J., Hillmann B., Lynch J., Sidiropoulos D., Spear J.R., Caporaso G., Blekhman R., Knight R. (2017). BugBase predicts organism-level microbiome phenotypes. bioRxiv.

[19] Gomez K.A., Gomez A.A. (1984). Statistical Procedures for Agricultural Research.

[20] Carter M.R., Gregorich E.G. (2008). Soil Sampling and Methods of Analysis.

[21] Zhou J., Bruns M.A., Tiedje J.M. (1996). DNA recovery from soils of diverse composition. Appl. Environ. Microbiol..

[22] Bao S.D. (2000). Soil and Agricultural Chemistry Analysis.

[23] Murphy J., Riley J.P. (1962). A modified single solution method for the determination of phosphate in natural waters. Anal. Chim. Acta.

[24] Olsen S.R., Cole C.V., Watanabe F.S., Dean L.A. (1954). Estimation of Available Phosphorus in Soils by Extraction with Sodium Bicarbonate.

[25] Knudsen D., Peterson G.A., Pratt P.F., Page A.L., Miller R.H., Keeney D.R. (1982). Lithium, sodium, and potassium. Methods of Soil Analysis: Part 2 Chemical and Microbiological Properties.

[26] Nelson D.W., Sommers L.E., Sparks D.L., Page A.L., Helmke P.A., Loeppert R.H., Soltanpour P.N., Tabatabai M.A., Johnston C.T., Sumner M.E. (1996). Total carbon, organic carbon, and organic matter. Methods of Soil Analysis: Part 3 Chemical Methods.

[27] Bremner J.M., Sparks D.L., Page A.L., Helmke P.A., Loeppert R.H., Soltanpour P.N., Tabatabai M.A., Johnston C.T., Sumner M.E. (1996). Nitrogen-total. Methods of Soil Analysis: Part 3 Chemical Methods.

[28] Caporaso J.G., Lauber C.L., Walters W.A., Berg-Lyons D., Huntley J., Fierer N., Owens S.M., Betley J., Fraser L., Bauer M. (2012). Ultra-high-throughput microbial community analysis on the Illumina HiSeq and MiSeq platforms. ISME J..

[29] Klindworth A., Pruesse E., Schweer T., Peplies J., Quast C., Horn M., Glöckner F.O. (2013). Evaluation of general 16S ribosomal RNA gene PCR primers for classical and next-generation sequencing-based diversity studies. Nucleic Acids Res..

[30] Bolger A.M., Lohse M., Usadel B. (2014). Trimmomatic: A flexible trimmer for Illumina sequence data. Bioinformatics.

[31] Magoč T., Salzberg S.L. (2011). FLASH: Fast length adjustment of short reads to improve genome assemblies. Bioinformatics.

[32] Edgar R.C. (2010). Search and clustering orders of magnitude faster than BLAST. Bioinformatics.

[33] Edgar R.C., Haas B.J., Clemente J.C., Quince C., Knight R. (2011). UCHIME improves sensitivity and speed of chimera detection. Bioinformatics.

[34] Caporaso J.G., Kuczynski J., Stombaugh J., Bittinger K., Bushman F.D., Costello E.K., Fierer N., Peña A.G., Goodrich J.K., Gordon J.I. (2010). QIIME allows analysis of high-throughput community sequencing data. Nat. Methods.

[35] Quast C., Pruesse E., Yilmaz P., Gerken J., Schweer T., Yarza P., Peplies J., Glöckner F.O. (2013). The SILVA ribosomal RNA gene database project: Improved data processing and web-based tools. Nucleic Acids Res..

[36] Wang Q., Garrity G.M., Tiedje J.M., Cole J.R. (2007). Naïve Bayesian classifier for rapid assignment of rRNA sequences into the new bacterial taxonomy. Appl. Environ. Microbiol..

[37] Chao A. (1984). Nonparametric estimation of the number of classes in a population. Scand. J. Stat..

[38] Schloss P.D., Westcott S.L., Ryabin T., Hall J.R., Hartmann M., Hollister E.B., Lesniewski R.A., Oakley B.B., Parks D.H., Robinson C.J. (2009). Introducing mothur: Open-source, platform-independent, community-supported software for describing and comparing microbial communities. Appl. Environ. Microbiol..

[39] Bray J.R., Curtis J.T. (1957). An ordination of the upland forest communities of southern Wisconsin. Ecol. Monogr..

[40] Legendre P., Legendre L. (2012). Numerical Ecology.

[41] Brown M.B., Forsythe A.B. (1974). Robust tests for the equality of variances. J. Am. Stat. Assoc..

[42] Shapiro S.S., Wilk M.B. (1965). An analysis of variance test for normality (complete samples). Biometrika.

[43] Kruskal W.H., Wallis W.A. (1952). Use of ranks in one-criterion variance analysis. J. Am. Stat. Assoc..

[44] Mann H.B., Whitney D.R. (1947). On a test of whether one of two random variables is stochastically larger than the other. Ann. Math. Stat..

[45] Clarke K.R. (1993). Non-parametric multivariate analyses of changes in community structure. Aust. J. Ecol..

[46] Benjamini Y., Hochberg Y. (1995). Controlling the false discovery rate: A practical and powerful approach to multiple testing. J. R. Stat. Soc. Ser. B Methodol..

[47] Borcard D., Legendre P., Drapeau P. (1992). Partialling out the spatial component of ecological variation. Ecology.

[48] Mantel N. (1967). The detection of disease clustering and a generalized regression approach. Cancer Res..

[49] Kirk G.J.D. (2004). The Biogeochemistry of Submerged Soils.

[50] Kögel-Knabner I., Amelung W., Cao Z., Fiedler S., Frenzel P., Jahn R., Kalbitz K., Kölbl A., Schloter M. (2010). Biogeochemistry of paddy soils. Geoderma.

[51] McDaniel M.D., Tiemann L.K., Grandy A.S. (2014). Does agricultural crop diversity enhance soil microbial biomass and organic matter dynamics? A meta-analysis. Ecol. Appl..

[52] Jackson R.B., Canadell J., Ehleringer J.R., Mooney H.A., Sala O.E., Schulze E.D. (1996). A global analysis of root distributions for terrestrial biomes. Oecologia.

[53] Fierer N. (2017). Embracing the unknown: Disentangling the complexities of the soil microbiome. Nat. Rev. Microbiol..

[54] Rousk J., Bååth E., Brookes P.C., Lauber C.L., Lozupone C., Caporaso J.G., Knight R., Fierer N. (2010). Soil bacterial and fungal communities across a pH gradient in an arable soil. ISME J..

[55] Trivedi P., Delgado-Baquerizo M., Trivedi C., Hu H., Anderson I.C., Jeffries T.C., Zhou J., Singh B.K. (2016). Microbial regulation of the soil carbon cycle: Evidence from gene–enzyme relationships. ISME J..

[56] Philippot L., Raaijmakers J.M., Lemanceau P., van der Putten W.H. (2013). Going back to the roots: The microbial ecology of the rhizosphere. Nat. Rev. Microbiol..

[57] Conrad R. (2007). Microbial ecology of methanogens and methanotrophs. Adv. Agron..

