# Supplementary material for: Is Early Tobacco–Rice Rotation Associated with Soil Nutrient Legacy and Shifts in Bacterial Ecological Potential in a Subtropical Paddy System?
Source: Plants (Basel). 2026 Jul 21;15(14):2230. doi: 10.3390/plants15142230 (PMC13417327; doi:10.3390/plants15142230)
Supplement: Supplementary file 1 [file plants-15-02230-s001.zip › plants-4417171-supplementary.pdf]

Supplementary Information

Table S1. Sampling hierarchy, statistical unit, and inferential scope.

Dataset	Recorded structure	Total n	Analysis unit	Permitted interpretation
Soil physicochemical properties	3 recorded field-composite observations per pathway-period-depth group	24	Field-composite observation	Descriptive/sample-level summaries; the early-period sample is not a common pretreatment baseline
16S rRNA profiles	6 profiles per pathway-period-depth group	48	Sequencing profile nested within pathway	Sample-level community configuration; not six independent fields
Agronomic traits except panicle number	4 recorded observations per pathway	8 per trait	Within-pathway measurement subsample	Descriptive only; quadrat-to-block identifiers unavailable
Panicle number	15 recorded observations per pathway	30	Within-pathway measurement subsample	Descriptive only; observation-to-block identifiers unavailable

Note: RR, rice-rice pathway; TR, tobacco-rice pathway.

Table S2. Beta-diversity ordination fit and global tests by soil depth.

Depth (cm)	Profiles (n)	Analysis	Statistic	Value	R^2	P value	q value	Interpretation
0-10	24	NMDS	Normalized stress	0.092810	NA	NA	NA	Ordination fit; not a significance test
10-20	24	NMDS	Normalized stress	0.092810	NA	NA	NA	Ordination fit; not a significance test
0–10	24	PERMANOVA	F	11.6305	0.6356	0.001	NA (omnibus test)	Four pathway-period groups; sample-level compositional pattern
10–20	24	PERMANOVA	F	6.6286	0.4986	0.001	NA (omnibus test)	Four pathway-period groups; sample-level compositional pattern
0–10	24	ANOSIM	R	0.8637	NA	0.001	NA (omnibus test)	Concordant sample-level separation
10–20	24	ANOSIM	R	0.7477	NA	0.001	NA (omnibus test)	Concordant sample-level separation
0–10	24	PERMDISP	F	1.6449	NA	0.357	NA (diagnostic test)	No detected heterogeneity of multivariate dispersion
10–20	24	PERMDISP	F	2.2896	NA	0.226	NA (diagnostic test)	No detected heterogeneity of multivariate dispersion

Note: NMDS, non-metric multidimensional scaling; PERMANOVA, permutational multivariate analysis of variance; ANOSIM, analysis of similarities; PERMDISP, permutational analysis of multivariate dispersions. Analyses used Bray-Curtis dissimilarity and characterize sample-level community patterns. NA, not applicable.

Table S3. Agronomic descriptive summary calculated from all recorded observations.

Trait	Unit	RR n	RR mean ± SD	TR n	TR mean ± SD	TR vs RR (%)	P value	q value
Grain yield	t ha^{-1}	4	6.58 ± 0.27	4	6.62 ± 0.38	+0.62	NA (descriptive)	NA (descriptive)
Panicle number	10^4 ha^{-1}	15	352.38 ± 51.07	15	392.06 ± 59.60	+11.26	NA (descriptive)	NA (descriptive)
Spikelets per panicle	count	4	149.88 ± 23.27	4	142.16 ± 5.79	-5.15	NA (descriptive)	NA (descriptive)
Seed-setting rate	%	4	60.28 ± 2.42	4	59.49 ± 4.18	-1.33	NA (descriptive)	NA (descriptive)
1000-grain weight	g	4	21.09 ± 0.63	4	22.18 ± 0.76	+5.18	NA (descriptive)	NA (descriptive)
Aboveground dry matter	t ha^{-1}	4	15.79 ± 3.32	4	18.23 ± 1.91	+15.47	NA (descriptive)	NA (descriptive)

Note: All recorded observations were retained; no value was excluded and no missing value was mean-imputed. For grain yield, spikelets per panicle, seed-setting rate, 1000-grain weight, and aboveground dry matter, n = 4 per pathway; for panicle number, n = 15 per pathway. Treatment-level P values, q values, and significance letters are not reported because the available records do not map measurement subsamples unambiguously to independent field blocks. SD, standard deviation; NA, not applicable.

Table S4. Alpha-diversity summaries using the 40 sequencing profiles plotted in Figure 2.

Depth (cm)	Pathway	Period	n	Chao1	Shannon	Simpson	Good's coverage	P value	q value
10-20	RR	Early	5	5714.9 ± 179.3	7.210 ± 0.057	0.00218 ± 0.00024	0.95378 ± 0.00196	NA (descriptive)	NA (descriptive)
10-20	RR	Late	5	5327.4 ± 53.8	7.169 ± 0.052	0.00247 ± 0.00049	0.95815 ± 0.00060	NA (descriptive)	NA (descriptive)
0-10	RR	Early	5	6195.3 ± 320.9	7.312 ± 0.049	0.00205 ± 0.00031	0.94903 ± 0.00375	NA (descriptive)	NA (descriptive)
10-20	TR	Late	5	5044.7 ± 208.5	7.125 ± 0.018	0.00243 ± 0.00018	0.96084 ± 0.00244	NA (descriptive)	NA (descriptive)
0-10	TR	Late	5	5033.0 ± 153.2	7.097 ± 0.011	0.00244 ± 0.00013	0.96037 ± 0.00203	NA (descriptive)	NA (descriptive)
0-10	RR	Late	5	5099.3 ± 123.2	6.988 ± 0.010	0.00293 ± 0.00016	0.95898 ± 0.00117	NA (descriptive)	NA (descriptive)
0-10	TR	Early	5	5499.2 ± 180.6	7.084 ± 0.060	0.00282 ± 0.00047	0.95519 ± 0.00235	NA (descriptive)	NA (descriptive)
10-20	TR	Early	5	5015.6 ± 402.1	6.956 ± 0.103	0.00309 ± 0.00030	0.95984 ± 0.00363	NA (descriptive)	NA (descriptive)

Note: Values are mean ± SD; n = 5 sequencing profiles per pathway-period-depth group. Figure 2 uses the 40 profiles retained by the figure-generation multimetric filtering step; the unfiltered source contains 48 profiles. Profiles are community-characterization units nested within the field comparison, so treatment-level P and q values are not reported. NA, not applicable.

Table S5. Contrast-ranked genus selection and late-period group means used in Figure 4E.

Contrast rank	Genus	Overall late mean	RR 0–10 cm	TR 0–10 cm	RR 10–20 cm	TR 10–20 cm	Pooled RR mean	Pooled TR mean
1	Thioalkalispira-Sulfurivermis	0.006683	0.000253	0.007602	0.002865	0.016011	0.001559	0.011807
2	Desulfobacca	0.007884	0.016301	0.004334	0.006759	0.004142	0.011530	0.004238
3	Candidatus_Solibacter	0.009110	0.003749	0.013451	0.007530	0.011709	0.005640	0.012580
4	Sulfuricurvum	0.003478	0.013120	0.000062	0.000460	0.000269	0.006790	0.000165
5	Arthrobacter	0.004043	0.000890	0.005275	0.001231	0.008776	0.001060	0.007026
6	Sphingomonas	0.005757	0.002239	0.007757	0.003589	0.009443	0.002914	0.008600
7	Gemmatimonas	0.005196	0.000977	0.007566	0.004499	0.007742	0.002738	0.007654
8	Bryobacter	0.007058	0.003812	0.010064	0.005792	0.008564	0.004802	0.009314

Note: Late-season genera were restricted to interpretable genus names, ranked first by the absolute RR-TR difference in mean relative abundance across depths and then by overall mean abundance, and the top eight were retained. The heatmap displays within-genus row z-scores. The listed group means reproduce the data used to generate Figure 4E.
